# Combined transcriptomics and metabolomics analyses reveal the molecular mechanism of heat tolerance in *Pichia kudriavzevii*

**DOI:** 10.3389/fmicb.2025.1572004

**Published:** 2025-04-09

**Authors:** Ning Wang, Lu Li, Yi Ma, Caihong Shen, Zonghua Ao, Chuan Song, Muhammad Aamer Mehmood, Puyu Zhang, Ying Liu, Xiaoke Sun, Hui Zhu

**Affiliations:** ^1^Sichuan Province Engineering Technology Research Center of Liquor-Making Grains, School of Bioengineering, Sichuan University of Science and Engineering, Yibin, China; ^2^Liquor Brewing Biotechnology and Application Key Laboratory of Sichuan Province, Yibin, China; ^3^National Engineering Research Center of Solid-State Brewing, Luzhou Laojiao Co., Ltd., Luzhou, China; ^4^Sichuan Yibin Hengshengfu Liquor Industry Group Co., Ltd., Yibin, China

**Keywords:** *Pichia kudriavzevii*, transcriptomics, metabolomics, high temperature stress, heat tolerance mechanism

## Abstract

**Introduction:**

*Pichia kudriavzevii* is a prevalent non-*Saccharomyces cerevisiae* yeast in *baijiu* brewing. The aim of this study was to isolate a high temperature resistant *Pichia kudriavzevii* strain from the *daqu* of strong flavor *baijiu* and to elucidate its molecular mechanism.

**Methods:**

Growth activity was assessed at temperatures of 37°C, 40°C, 45°C, and 50°C. Morphological changes were observed by scanning electron microscopy at 37°C, 45°C, and 50°C. Subsequent analysis of the transcriptomics and metabolomics was undertaken to elucidate the molecular mechanism of heat tolerance.

**Results:**

The strain was able to tolerate high temperature of 50°C, undergoing substantial morphological alterations. Gene ontology (GO) analysis of the transcriptomics revealed that differentially expressed genes (DEGs) were enriched in pathways such as ATP biosynthesis process and mitochondrion; Kyoto Encyclopedia of Genes and Genomes (KEGG) pathway enrichment analysis showed that DEGs were up regulated in oxidative phosphorylation. Utilising liquid chromatograph-mass spectrometer, a total of 463 cationic differential metabolites and 352 anionic differential metabolites were detected and screened for differential substances that were closely related to heat tolerance (NAD+ and ADP); KEGG analysis showed that metabolites were up regulated in purine metabolism. Furthermore, correlation analyses of transcriptomics-metabolomics demonstrated a strong positive correlation between the metabolites NAD+ and ADP, and multiple DEGs of the oxidative phosphorylation pathway.

**Discussion:**

These results suggest that the heat tolerant strain can be able to counteract high temperature environment by up regulating energy metabolism (especially oxidative phosphorylation) to increase ATP production.

## 1 Introduction

*Baijiu* is an ancient distilled spirit that originated in China and has a history of more than 2,000 years. In comparison with foreign distilled spirits, there is a significant disparity in the raw materials and technology utilised in its production (Liu and Sun, 2018). It is typically produced through the fermentation of a blend of sorghum, rice, corn, barley, wheat, and other grain crops, following specific combinations and ratios (Kang et al., 2023). This fermentation process entails a complex microbial activity (Tu et al., 2022), wherein the microbiota, through its own succession and growth patterns, and metabolic processes, generates a multitude of fermentation metabolites that collectively contribute to the formation of the distinctive flavor profile of *baijiu* (Zhang et al., 2024). *Daqu* is a saccharification fermenter employed in the production of *baijiu*, and it is distinguished by its rich microbial communities, functional enzyme systems, and flavor precursors (Li et al., 2023). The classification of *daqu* utilised in the context of *baijiu* fermentation can be predicated on the processing temperature range: high temperature (60–70°C), medium-high temperature (55–60°C), medium-temperature (50–55°C), and low-temperature (40–50°C) (Zhang et al., 2024; Zhou et al., 2022). Therefore, the temperature of the microorganism growth environment frequently exceeds 40°C, particularly in high temperature *daqu*, and the microbial population decreases as the temperature rises (Gou et al., 2015; Wang et al., 2008). Indeed, in the process of producing *baijiu*, the microbiological system is frequently exposed to extreme conditions, including high temperature, high acidity, and high ethanol level (Wang N. et al., 2023). Notably, high temperature has been identified as a pivotal factor influencing the activity of brewing microorganisms, the rate of microbial growth, and the production of metabolites (Amillastre et al., 2012; Mejía-Barajas et al., 2017). It has been established that high temperature can induce hazards such as diminished cell viability, inhibition of the growth rate, disruption of cellular and mitochondrial membranes (Richter et al., 2010).

For many years, *Saccharomyces cerevisiae* has been considered the most prevalent yeast in the fermentation process of *baijiu*, and it is the microorganism that exerts a substantial influence on the fermentation process (Hu et al., 2021). Indeed, non-*Saccharomyces cerevisiae* yeast has been shown to play an essential role in the brewing process (Gou et al., 2015). It has been established that certain non-*Saccharomyces cerevisiae* yeasts possess remarkable heat tolerance and are capable of adapting to the high temperature encountered during *baijiu* fermentation (Xiong et al., 2014). Consequently, the isolation and utilisation of heat tolerant non-*Saccharomyces cerevisiae* yeast during *baijiu* brewing can contribute to the stabilisation of the ecological balance of brewing microorganisms under extreme high temperature conditions. *Pichia kudriavzevii* is a yeast that is found in nature and is frequently employed in the fermentation of foodstuffs due to its ability to withstand high temperature, acid, and ethanol (Chu et al., 2023; Zhou et al., 2022). *Pichia kudriavzevii* is the dominant non-*Saccharomyces cerevisiae* yeast in a wide range of aroma types of *baijiu*, including strong flavor, sauce flavor, and light flavor (Wang et al., 2019). During the initial phase of *baijiu* fermentation, a greater diversity of yeast species is present. However, as the fermentation process continues at high temperature, there is a notable decline in the number and species of yeast, but *Pichia kudriavzevii* maintains predominant at high temperature (You et al., 2021). The majority of *Pichia kudriavzevii* are capable of growth within the temperature range of 25–40°C, and it has been demonstrated that *Pichia kudriavzevii* exhibits greater heat tolerance than *Saccharomyces cerevisiae* (Wang et al., 2024).

In this study, a *Pichia kudriavzevii* strain was isolated from the *daqu* of strong flavor *baijiu*, which was found to have good heat tolerance by measuring growth activity. Utilising transcriptomics and metabolomics, we sought to elucidate the molecular underpinnings of the strain’s heat tolerance, with the objective of providing an idea for the investigation of heat tolerance mechanisms in *Pichia kudriavzevii*, as well as to develop methodologies for the control and optimisation of the *baijiu* brewing process.

## 2 Materials and methods

### 2.1 Screening of heat tolerant yeast strain

We isolated some heat tolerant strains from *daqu* of strong flavor *baijiu*, and the *daqu* sample was obtained from a *baijiu* distillery in Yibin, China. Subsequently, the most heat tolerant strain was selected and identified as a strain of *Pichia kudriavzevii*. The isolate has been registered with the China General Microbiological Culture Collection Center (CGMCC), and the accession number (CGMCC No. 33254). To evaluate the heat tolerance potential of the strain, a YPD liquid medium (Sangon Biotech, Shanghai, China) was prepared. The strain growth temperature was adjusted by setting different incubator temperatures, including 37°C, 40°C, 45°C, and 50°C. The 37°C was set as the control. Then, the samples were inoculated in a 48-well plate at 5% (v/v) inoculum, and the well plates were placed in a MicroScreen-HT real-time microbial growth analysis system (Gering, Tianjin, China). The plate was incubated at 180 rpm with shaking, and the OD_600_ was measured.

### 2.2 Observation on morphological changes of heat tolerant yeast

The methods are based on the previous report with some modifications (Wang et al., 2024). The strains were incubated at various temperatures (37°C, 45°C, and 50°C), with 37°C serving as the control group. Following a 48-h incubation period, the samples were washed 3 times with 0.1 M PBS (pH 7.0), fixed with 5.4% (v/v) glutaraldehyde at 4°C for 24 h. The fixative was discarded and the sample was rinsed with 0.1 M PBS (pH 7.0) for 3 times (15 min each time). Subsequently, the samples were dehydrated with different concentrations of ethanol (50, 60, 70, 80, 90, and 100%) for 15–20 min. The dehydrated samples were transferred to a mixture of ethanol and isoamyl acetate (v:v = 1:1) for about 30 min, then to pure isoamyl acetate for about 1 h. Finally, the samples were dried at critical point with liquid CO_2_, coated with palladium and observed under scanning electron microscopy (SEM; VEGA3 TESCAN, Tescan, Brno, Czech Republic).

### 2.3 RNA samples preparation, extraction, and sequencing

The strain was inoculated in YPD liquid medium (50 mL). Growth temperatures were adjusted to 37°C (C group) and 45°C (HT group). Then, the samples were inoculated at 5% (v/v) and shaken at 180 rpm. As indicated by the growth curves, the samples were collected from each group at the midpoint of the logarithmic growth period. The samples were then subjected to a centrifugal process at 3,000 rpm for 3 min, after which the resultant supernantants were discarded. The samples were then immediately placed into liquid nitrogen for a period of 10 min, after which they were stored at a temperature of −80°C.

Samples from the high temperature group (HT) and the control group (C) were collected for RNA extraction and three replicates were set up for each group. Total RNA was extracted using a Trizol kit (Invitrogen, Carlsbad, CA, United States). RNA quality and quantity were then determined, libraries were constructed and sequenced using the Illumina Novaseq platform to obtain paired-end reads of 150 bp.

### 2.4 Transcriptomics analysis

The transcriptomics analysis part was conducted by Novogene Co. (Beijing, China). The DESeq2 R package (1.20.0) was used to compare the differential expression analyses between groups, and the clusterProfiler R package (3.8.1) was used to perform Gene Ontology (GO) enrichment analyses of the differentially expressed genes (DEGs) and to correct for gene length bias. The Kyoto Encyclopedia of Genes and Genomes (KEGG) enrichment analysis of DEGs was performed using clusterProfiler R package (3.8.1). In order to address the issue of the false positive rate resulting from multiple comparisons, the Benjamini–Hochberg method was employed to recalibrate the *p*-value and obtain *p*-adj, thereby ensuring the control of the false discovery rate (FDR). The screening of DEGs was conducted based on the absolute values of |log_2_ (FoldChange)| >1 and *p*-adj <0.05. DEGs were undertaken for GO enrichment and KEGG pathway enrichment, with the screening condition of *p*-adj <0.05.

### 2.5 Untargeted metabolomics analysis of heat tolerant yeast

Untargeted metabolomics analysis was performed using liquid chromatograph-tandem mass spectrometer (LC-MS/MS). Metabolomics analyses were performed on a Vanquish UHPLC (Thermo Fisher, Germany) equipped with a Hypersil Gold column (C18) (100 × 2.1 mm, 1.9 μm, Thermo Fisher, United States) coupled to Q Exactive^™^ HF/Q Exactive^™^ HF-X (Thermo Fisher, Germany) in Novogene Co., Ltd. (Beijing, China). Mobile phase A was 0.1% FA in water, while mobile phase B was methanol. The flow rate was 0.2 mL/min, and the column temperature was 40°C. The gradient elution program was as follows: 0–1.5 min, 2% B; 1.5–3 min, 2 to 85% B; 3–10 min, 85 to 100% B; 10–10.1 min, 100 to 2% B; 10.1–12 min, 2% B. Q Exactive^TM^ HF mass spectrometer was operated in positive/negative polarity mode with spray voltage of 3.5 kV, capillary temperature of 320°C, sheath gas flow rate of 35 psi and aux gas flow rate of 10 L/min, S-lens RF level of 60, Aux gas heater temperature of 350°C. Peak detection, molecular formula fitting, and peak area extraction were performed on the raw data using Compound Discoverer 3.3 (CD 3.3, Thermo Fisher). Principal component analysis (PCA) and partial least squares discriminant analysis (PLS-DA) were performed using the metaX (a flexible and comprehensive software for processing metabolomics data). The differential metabolites were screened according to the criteria of VIP >1.0, FC >1.5 or FC <0.667, and *p*-value <0.05. The differential metabolites were undertaken for KEGG pathway enrichment, with the screening condition of *p*-value <0.05.

### 2.6 Statistical analysis

The analysis of the data was conducted using GraphPad Prism 9.0 (San Diego, CA, United States), and the resulting data sets were visualised through the creation of graphs. In consideration of the divergent requirements of sequencing platforms for the screening of differential genes and differential metabolites, it was determined that *p*-adj would serve as a metric of significant difference in transcriptomics, and *p*-value in metabolomics. The specific screening criteria are shown above.

## 3 Results

### 3.1 Analysis of high temperature resistance of heat tolerant yeast

**Figure 1:**
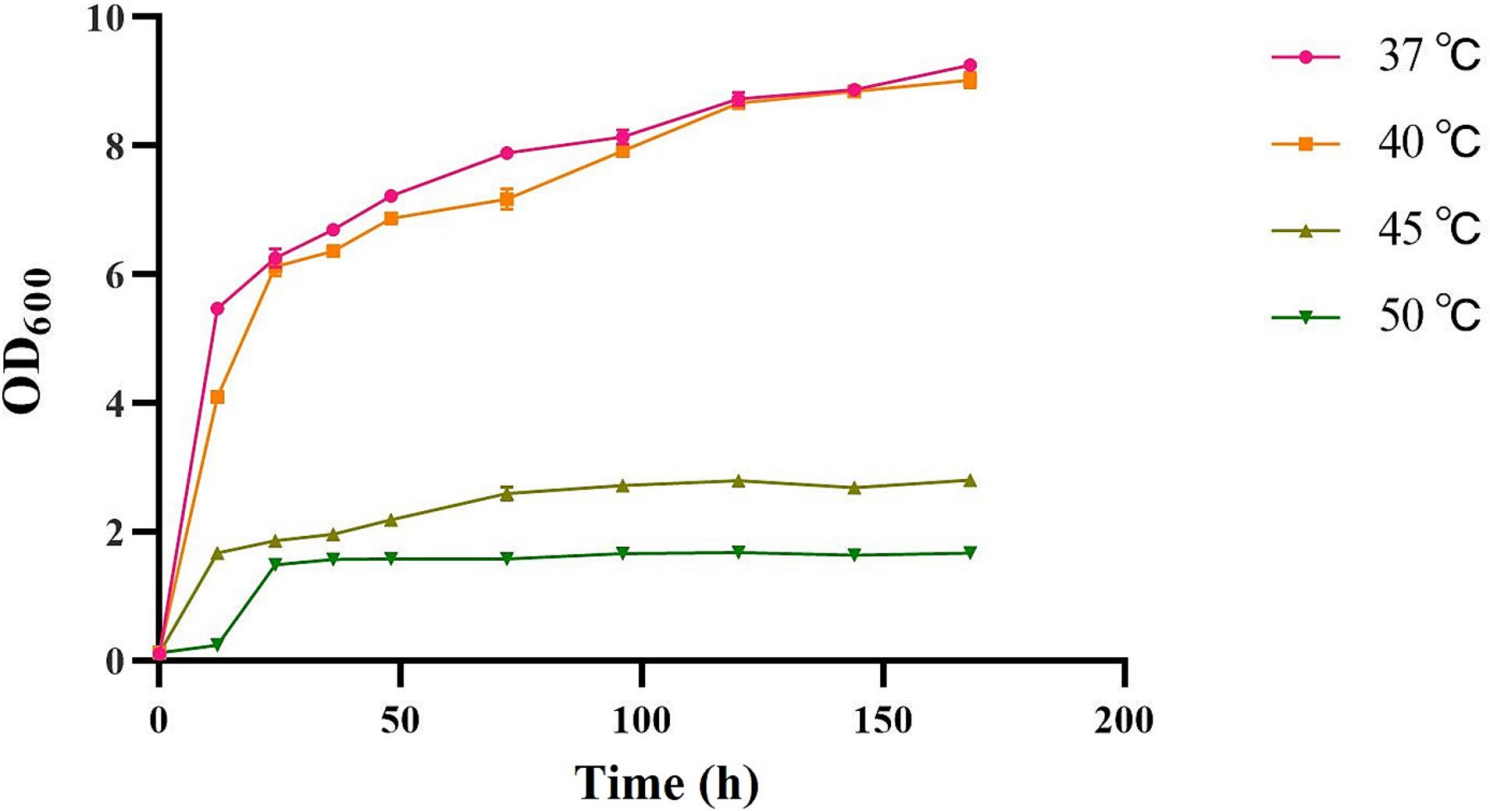
The OD_600_ of heat tolerant yeasts at different incubation temperatures (37°C, 40°C, 45°C, and 50°C).

The strains were incubated at different temperatures, including 37°C (control), 40°C, 45°C, and 50°C. It was found that the strains proliferated rapidly during the logarithmic growth period at 37°C and 40°C. The results showed that varying incubation temperatures exerted a substantial influence on the OD_600_ of the strain (Figure 1). As the temperature continued to increase, the strain was still able to grow, although the growth rate and state decreased, indicating that the strain was able to tolerate the high temperature of 50°C, but the growth state of the strain at 45°C was better than that at 50°C. In order to successfully carry out the subsequent molecular mechanism investigation, we chose 45°C as the high temperature treatment condition.

### 3.2 Morphological and structural changes of heat tolerant yeast

**Figure 2:**
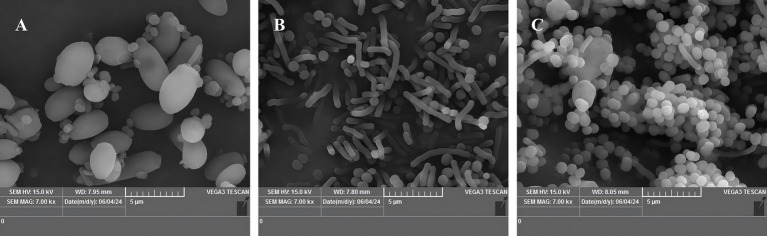
Scanning electron micrographs of strains at different incubation temperatures (7.00 k×). **(A)** Incubation at 37°C (control), **(B)** incubation at 45°C, **(C)** incubation at 50°C.

Changes in the morphology and structure of the strains cultivated at varying temperatures for 48 h were observed using scanning electron microscopy (Figures 2A–C). The results showed that sample A (37°C, control group), the body was fuller; sample B (45°C) compared to A, the morphology changed significantly, the body became significantly longer, in the form of a long rod; sample C (50°C) compared to A, the body became significantly smaller, in the form of round ball.

### 3.3 Transcriptomics analysis of heat tolerant yeast under high temperature stress

**Figure 3:**
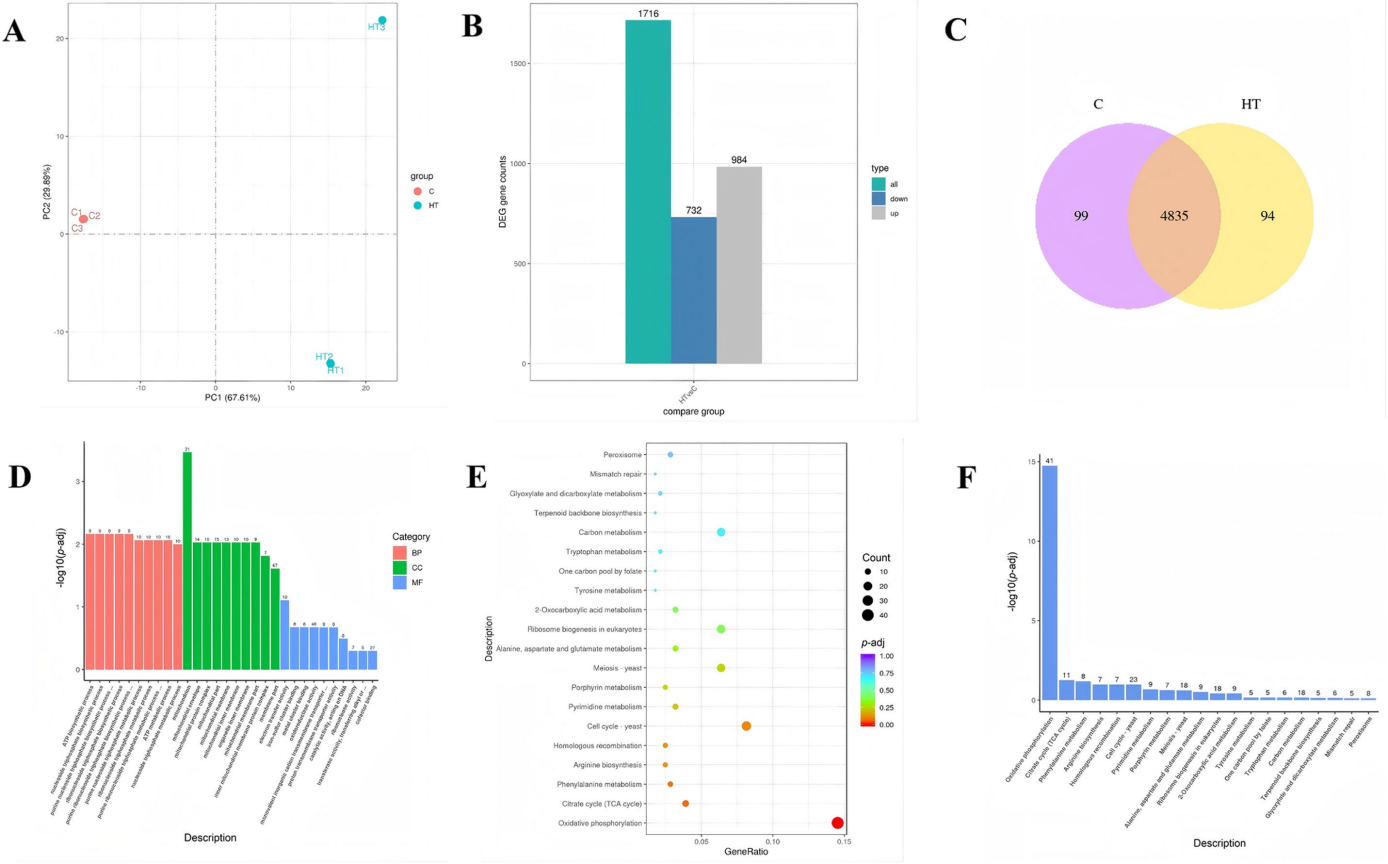
Transcriptomics analysis of heat tolerant yeast under high temperature stress (HT vs. C). C: control group (37°C), HT: high temperature group (45°C). **(A)** Principal component analysis (PCA) results. **(B)** Statistical histogram of the number of differential genes (DEG) in the differential comparison. **(C)** Co-expression Venn diagram. **(D)** Histogram of the GO up regulated enrichment analysis. The horizontal coordinate in the graph is the GO term and the vertical coordinate is the significance level of the GO term enrichment, expressed as −log_10_ (*p*-adj). −log_10_ (*p*-adj) represents the negative log_10_ transformation of the *p*-adj, highlighting the statistical significance of differential expression. Higher values indicate greater significance. The numbers on top of the bars denote the count of differentially expressed genes enriched in each GO term. **(E)** Scatter plot of the KEGG up regulated enrichment. The horizontal coordinate of the graph is defined as the ratio of the number of DEGs annotated to the KEGG pathway to the total number, and the vertical coordinate is represented by the KEGG pathway. **(F)** Bar graph of the KEGG up regulated enrichment analysis. The horizontal coordinate of the graph represents the KEGG pathway, while the vertical coordinate denotes the significance level of pathway enrichment. The numbers on top of the bars denote the count of differentially expressed genes enriched in each KEGG pathway.

According to the results of the previous analysis, the strain exhibits better growth at a high temperature of 45°C. Therefore, this temperature was selected as the high temperature culture conditions of the strain. In total, 280,839,674 raw reads were generated, and 276,634,950 clean reads were obtained after quality control filtering. Principal component analysis (PCA) is performed on the gene expression values (FPKM) of all samples in order to assess the differences between groups and the reproducibility of samples within groups. The results show that the samples are more dispersed between the C and HT groups, but more concentrated within the same group, suggesting a significant difference in FPKM between the two groups (Figure 3A). In addition, the combined change in the first and second principal components was 97.5% (PC1: 67.61%, PC2: 29.89%), indicating that there were differences between the C and HT groups. Compared with group C, a total of 1716 DEGs were identified under 45°C high temperature stress (HT group), of which 732 genes were down regulated and 984 genes were up regulated (Figure 3B). In addition, 99 and 94 genes were unique to the C and HT groups, respectively, while 4,835 genes were shared (Figure 3C).

DEGs were analysed for Gene Ontology (GO) enrichment. The results of the HT vs. C showed that the DEGs significantly up-regulated in biological processes (BP) were involved in ATP biosynthetic process (GO:0006754), nucleoside triphosphate biosynthetic process (GO:0009142), and purine nucleoside triphosphate biosynthetic process (GO:0009145), whereas DEGs significantly enriched in cellular component processes (CC) were involved in mitochondrion (GO:0005739), mitochondrial envelope (GO:0005740), and mitochondrial protein complex (GO:0098798) (Figure 3D and Supplementary Table S1). Consequently, the strain can enhance tolerance to high temperature by increasing the synthesis of energetic substances such as ATP.

As demonstrated in Figures 3E,F, KEGG pathway enrichment analysis showed that the pathways that were up regulated in the HT vs. C group predominantly associated with oxidative phosphorylation (ppa00190) and citrate cycle (TCA cycle) (ppa00020). Among these, the oxidative phosphorylation pathway exhibited a significant up regulation. This result lends further credence to the hypothesis that the thermotolerance mechanism of this strain is predominantly associated with the process of energy substance (e.g., ATP) production.

A detailed investigation into the DEGs associated with oxidative phosphorylation revealed that genes related to NADH dehydrogenase, complex I (NADH-ubiquinone oxidoreductase), complex II (succinate dehydrogenase), complex IV (cytochrome c oxidase), and cytochrome c were significantly up-regulated in the HT group (Table 1). In conditions of high temperature, the heat tolerant yeast predominantly regulates the oxidative phosphorylation pathway through the up regulation of the gene expression levels of key enzymes.

**Table 1:** DEGs related to oxidative phosphorylation.

Enzyme	Gene ID	Gene name	log_2_ (FoldChange)
NADH dehydrogenase	31692085	BOH78_1481	2.93
	31691434	BOH78_0821	4.33
	31694968	BOH78_4391	2.93
	31691405	BOH78_0791	1.02
	31695151	BOH78_4575	2.48
	31694682	BOH78_4104	1.81
	31690876	BOH78_0258	2.05
	31690921	BOH78_0303	1.12
	31693301	BOH78_2704	1.40
NADH-ubiquinone oxidoreductase	31691135	BOH78_0518	1.84
	31693254	BOH78_2657	2.34
	31693333	BOH78_2736	1.48
	31694986	BOH78_4409	3.04
	31693653	BOH78_3058	1.33
	31690694	BOH78_0074	2.63
	31692487	BOH78_1885	1.53
	31695404	BOH78_4834	1.02
Succinate dehydrogenase	31691967	BOH78_1362	1.44
Cytochrome c oxidase	31693261	BOH78_2664	2.31
	31694043	BOH78_3453	2.61
	31695036	BOH78_4459	1.95
	31695247	BOH78_4673	1.61
	31693265	BOH78_2668	1.61
	31694370	BOH78_3787	1.12
	31693546	BOH78_2951	2.16
	31692571	BOH78_1969	1.37
	31691564	BOH78_0951	1.19
	31692078	BOH78_1474	1.57
Cytochrome c	31690990	BOH78_0372	3.19

### 3.4 Metabolomics analysis of heat tolerant yeast under high temperature stress

**Figure 4:**
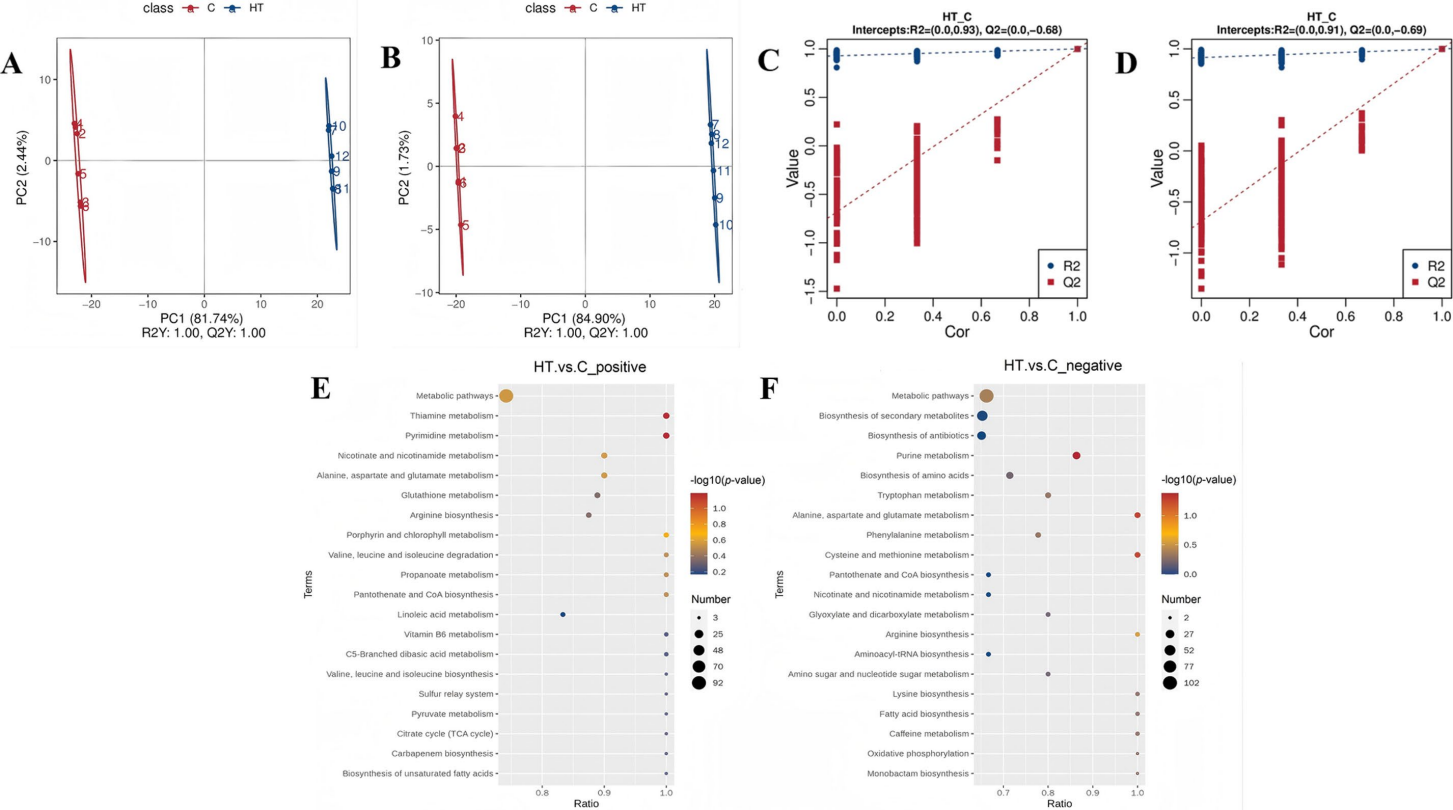
Metabolomics analysis of heat tolerant yeast under high temperature stress (HT vs. C). C: control group (37°C), HT: high temperature group (45°C). **(A)** PLS-DA score scatterplot_positive. **(B)** PLS-DA score scatterplot_negative. **(C)** PLS-DA ranked validation plot_positive. **(D)** PLS-DA ranked validation plot_negative. **(E)** Bubble plot of KEGG pathway enrichment analysis (HT vs. C_positive). **(F)** Bubble plot of KEGG pathway enrichment analysis (HT vs. C_negative). In the PLS-DA score scatterplot **(A,B)**, the horizontal coordinates represent the scores of the samples on the first principal component, while the vertical coordinates denote the scores of the samples on the second principal component. *R*^2^*Y* signifies the explanatory rate of the model, and *Q*^2^*Y* is employed to evaluate the predictive ability of the PLS-DA model. When the values of *R*^2^*Y* and *Q*^2^*Y* are close to 1, it indicates that the model is more stable and reliable. In the PLS-DA ranked validation plot **(C,D)**, the horizontal coordinate represents the correlation between the sample labeling information Y of the random grouping and the sample labeling information Y of the original grouping, and the vertical coordinate represents the scores of *R*^2^ and *Q*^2^. When the *R*^2^ data is greater than the *Q*^2^ data and the intercept of the *Q*^2^ regression line with the Y-axis is less than 0, it can be shown that the model is not “overfitted,” which means the model is feasible.

In order to identify the differential metabolites, the partial least squares discrimination analysis (PLS-DA) was utilised to compare the metabolite changes in this heat tolerant yeast following exposure to high temperature stress. The results of the PLS-DA analysis show that the two sample groups are more dispersed between groups, while clustering is called within groups. And both *R*^2^*Y* and *Q*^2^*Y* are equal to 1, which shows the reliability of the model. A significant separation between the samples from the two groups was observed in the horizontal direction, indicating a substantial difference in their metabolite compositions (Figures 4A,B). The *R*^2^ (0.93) and *Q*^2^ (−0.68) values for the positive polarity mode, and the *R*^2^ (0.91) and *Q*^2^ (−0.69) values for the negative polarity mode. It has been determined that the *R*^2^ data exceeds the *Q*^2^ data in both detection modes, and the intercept between the *Q*^2^ regression line and the Y-axis is less than 0. This indicates that there is no “overfitting” of the model, and the model is stable and feasible (Figures 4C,D).

The results demonstrated that there were 463 cationic metabolites with significant differences, of which 214 metabolites were up regulated and 249 metabolites were down regulated, and there were 352 anionic metabolites with significant differences, of which 116 metabolites were up regulated and 236 metabolites were down regulated. Among the metabolites that were found to be significantly up-regulated were those associated with heat tolerance, including nicotinamide adenine dinucleotide (NAD^+^), adenosine diphosphate (ADP), betaine, and adenosine 3′5′-cyclic monophosphate (Supplementary Table S2). Of particular note are NAD^+^ and ADP, which are closely related to oxidative phosphorylation and energy metabolism, thus suggesting a possible regulatory pathway for this heat tolerant yeast confronting high temperature stress.

The annotation of differently expressed cationic metabolites revealed their association with 44 metabolic pathways, including pyrimidine metabolism and thiamine metabolism (Figure 4E). In the case of differentially expressed anionic metabolites, their annotation pointed to 45 metabolic pathways, encompassing purine metabolism, cysteine, and methionine metabolism, alanine, aspartate and glutamate metabolism (Figure 4F). Of particular note was the significant enrichment of purine metabolism (*p-*value <0.05).

### 3.5 Transcriptomics-metabolomics association analysis of heat tolerant yeast under high temperature stress

**Figure 5:**
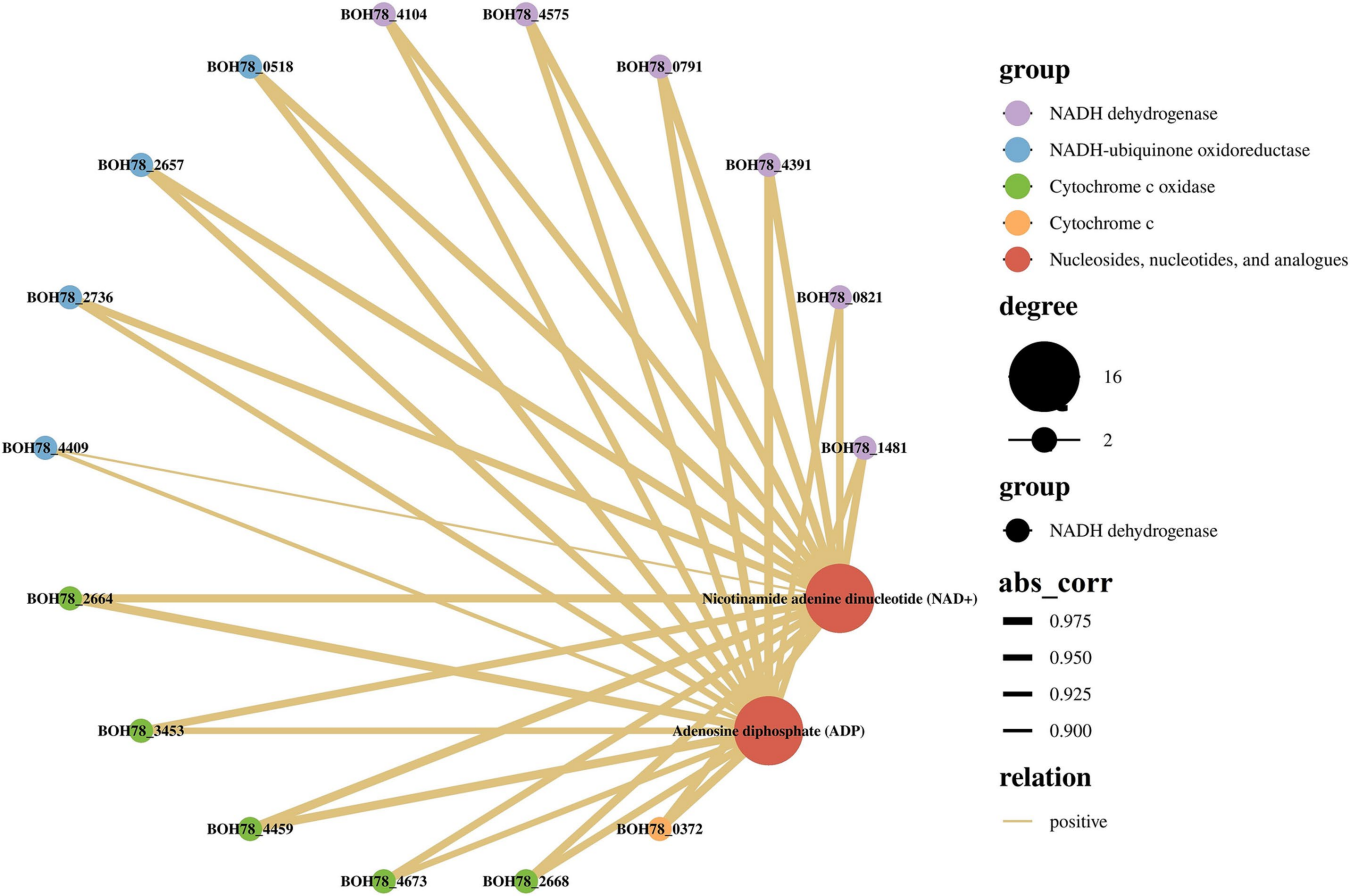
Transcriptomics-metabolomics association analysis in oxidative phosphorylation and energy metabolism (HT vs. C). C: control group (37°C), HT: high temperature group (45°C). The abs_corr refers to the absolute value of the correlation coefficient, which is a quantitative measure of the strength of the relationship between gene expression and metabolite levels. A higher abs_corr value (closer to 1) indicates a stronger correlation, while a lower value (closer to 0) suggests a weaker relationship. The larger the abs_corr coefficient, the thicker the line.

In order to provide a more accurate reflection of the relationship between genes and metabolites in this heat tolerant yeast, based on the results of the analyses above, the focus here was on the role of energy metabolism in the regulation of heat tolerance in the strain. Nicotinamide adenine dinucleotide (NAD^+^) and adenosine diphosphate (ADP) were consequently selected as the focal metabolites, and correlation analyses were conducted with oxidative phosphorylation-related DEGs in Table 1. A transcriptomics-metabolomics correlation network was then constructed (Figure 5). The DEGs associated with NADH dehydrogenase, NADH-ubiquinone oxidoreductase, cytochrome c oxidase, and cytochrome c in the oxidative phosphorylation pathway demonstrated a significant positive correlation with NAD^+^ and ADP. Collectively, these differential metabolites, associated with ATP production and energy metabolism in the metabolomics, exhibited a positive correlation with oxidative phosphorylated DEGs in the transcriptomics. This finding further supports the hypothesis that this heat tolerant yeast possesses the capacity to counteract high temperature by up regulating energy metabolism (especially oxidative phosphorylation). This process can constitute a pivotal mechanism through which the strain exhibits heat tolerance.

## 4 Discussion

The process of *baijiu* brewing frequently occurs in conditions characterised by high temperature, high acidity, and significant ethanol content. It is widely acknowledged that temperature is a pivotal factor in the fermentation process (Liu et al., 2023). The impact of fermentation temperature extends beyond the categorisation and quality of *baijiu*, also influencing the composition of the microbial community within the brewing process (Tu et al., 2022; Zhang et al., 2024). *Pichia kudriavzevii* plays a pivotal role in the brewing process of *baijiu* and is a dominant functional yeast in the fermentation process of typical *baijiu* (Chu et al., 2023; Li et al., 2013). Not only does it contribute to the establishment of the *baijiu* fermentation microbial community by influencing its dynamics during the fermentation process (Zhang et al., 2021), but it also enhances the ester content of *baijiu* and improves its flavor quality (Li et al., 2022). In addition, *Pichia kudriavzevii* was observed to be highly heat resistant, with existing studies demonstrating that temperatures ranging from 37 to 42°C significantly stimulated its growth during fermentation of sauce flavor *baijiu* (Zhang et al., 2021), and the yeast rapidly dominated the fermentation process at these temperatures. Furthermore, it was determined that *Pichia kudriavzevii* MBY1358 exhibited the capacity to withstand temperature of 44°C (Choi et al., 2017).

In this paper, a *Pichia kudriavzevii* strain that exhibited excellent heat tolerance was isolated from a strong flavor *baijiu* macroquat. The strain was cultivated at varying temperatures in order to ascertain its growth activity and to observe its morphological characteristics. In order to explore the molecular mechanism of heat resistance, transcriptomics, and metabolomics were employed. The findings of the present study demonstrated that the strain exhibited a high degree of temperature tolerance, with the capacity to withstand temperatures of up to 50°C. Furthermore, the incubation of the strain at elevated temperatures resulted in a substantial alteration of its morphological characteristics. It has been demonstrated by several studies that the morphology of strains frequently undergoes alterations in order to cope with high temperature damage subsequent to exposure to high temperature (Shen and Chou, 2016; Xiao et al., 2022). A study reported that heat stress (42°C) led to the development of a circular structure in *Saccharomyces cerevisiae* that differed from bud scars, with a twofold increase in cell wall stiffness (Pillet et al., 2014), which is related to heat stress-induced thickening of the yeast cell wall and alteration of cell membrane fluidity and the development of cell membrane fluidity (Lin et al., 2022). Furthermore, elevated temperatures have been observed to induce substantial internal structural changes in yeast, including a reduction in the size of the inner core (Webster and Watson, 1993) and alterations in yeast morphology due to changes in organelle size (Keuenhof et al., 2021). These results provide further evidence to support the hypothesis that yeast possess the capacity to counteract deleterious effects of elevated temperatures by undergoing alterations to their morphological characteristics. It is therefore hypothesised that the examined this heat tolerant *Pichia kudriavzevii* can exhibit resistance to thermal damage caused by heat stress through alterations in their morphology.

The transcriptomics analysis of the strain RNA samples revealed that the KEGG pathway enrichment analysis indicated up regulation of oxidative phosphorylation (*p*-adj <0.05) and citrate cycle (TCA cycle). These results suggested that the strain could counteract high temperature stress by enhancing oxidative phosphorylation and increasing energy metabolism, which might be the heat tolerance mechanism of the strain. Oxidative phosphorylation is considered to be one of the key activities of the protein complexes in the inner membrane of mitochondria, which is also viewed as the primary pathway for ATP synthesis within the mitochondria and a major source of energy in cellular pathways (Cooper and Hausman, 2007; van der Bliek et al., 2017). It has been established that during oxidative phosphorylation, electrons from NADH and FADH_2_ bind to O_2_. The energy released from these oxidation/reduction reactions is used to drive the synthesis of ATP from ADP (Cooper and Hausman, 2007). In response to altered environmental conditions, fungi have been observed to modify their energy metabolism pathways, thereby acclimatising to the novel conditions (Marcet-Houben et al., 2009). A multi-omics analysis of the mechanism of high temperature tolerance in *Kluyveromyces marxianus* was conducted, and oxidative phosphorylation was identified as a significant enrichment pathway. These findings suggest that this yeast possesses the capacity to counteract high temperature damage by regulating the oxidative phosphorylation pathway (Kosaka et al., 2022; Li et al., 2019). In addition to yeast, a proteomic analysis of *Aspergillus niger* under 50°C treatment conditions revealed significant changes in various proteins within the oxidative phosphorylation KEGG pathway (Deng et al., 2020). In addition, the TCA cycle has been demonstrated to be associated with microbial heat tolerance, as well as oxidative phosphorylation. The TCA cycle represents a pivotal energy metabolic pathway, serving as the central pathway of cellular oxidative phosphorylation, which generates NADH and FADH_2_ (Anderson et al., 2018; Shih and Pan, 2011). A study was conducted to perform a transcriptomics analysis for the purposes of investigating the heat tolerance exhibited by *Pichia kudriavzevii* LC375240, and the results obtained revealed that there was a significant up regulation of genes belonging to the TCA cycle pathway in the strain that had been subjected to heat treatment (Qi et al., 2025).

In this paper, LC-MS/MS was utilised to analyse the metabolite changes in heat tolerant yeast following exposure to high temperature stress. The study identified a total of 463 differential metabolites in the positive polarity mode and 352 differential metabolites in the negative polarity mode. Among these, the differential metabolites related to heat tolerance were found to be significant, including a significant up regulation of nicotinamide adenine dinucleotide (NAD^+^) and adenosine diphosphate (ADP). These substances are closely related to oxidative phosphorylation and energy metabolism. NAD^+^ is a vital pyridine nucleotide cofactor that plays a pivotal role in various aspects of cellular respiration and energy production. It is employed in the synthesis of ATP and the maintenance of membrane potential in mitochondria (Massudi et al., 2012). NAD^+^ is a cofactor that plays an essential role in a variety of significant biochemical processes within cells, including oxidative phosphorylation, ATP production, DNA repair, calcium-dependent secondary messengers (Braidy et al., 2019; Gindri et al., 2024). In the context of aerobic eukaryotic cells, the predominant pathway for the production of the energy metabolite ATP is through mitochondrial oxidative phosphorylation. The mitochondrial ADP/ATP carrier plays a pivotal role in this process by facilitating the input of ADP into the cytoplasmic lysosome and the export of ATP from the mitochondrial matrix. This process represents a pivotal step in the broader mechanism of oxidative phosphorylation within the context of eukaryotic cells (Clémençon et al., 2013; Kunji et al., 2016). In addition to substances associated with energy metabolism, a variety of other substances have been linked to increased heat resistance, including betaine and adenosine 3′5′-cyclic monophosphate. Betaine is a compound that is found in a wide variety of organisms, including bacteria, fungi, higher plants and animals. Microorganisms have been observed to utilise betaine as a stress protector against a range of environmental challenges, such as temperature stress, drought and other forms of stress (Mo et al., 2022). As a second messenger, cAMP has been demonstrated to exert a pivotal function in a broad spectrum of physiological responses in bacteria, fungi, and plants and animals (Liang et al., 2022), and can modulate the response of organisms to extreme environments such as heat stress and osmotic stress (Zhu et al., 2016).

Further KEGG pathway enrichment analysis of the differential metabolites revealed that the metabolites were enriched in purine metabolism (*p*-value <0.05), pyrimidine metabolism, and multiple amino acid metabolism (cysteine, methionine, alanine, aspartate, and glutamate, etc.) pathways. Purine metabolites, most notably purine nucleotides, function not only as building blocks for DNA and RNA, but also as vital sources of cellular energy (e.g., ATP) and cofactors that promote cell survival and proliferation (Tran et al., 2024). Furthermore, they play a crucial role in intracellular and intercellular signalling pathways (Gessner et al., 2023; Pedley and Benkovic, 2017; Wang L. et al., 2023). It has been demonstrated that the purine metabolism pathway in yeast exerts a dual regulatory effect on ATP levels and NAD^+^ synthesis. When purine metabolism is up regulated, there is an increase in intracellular ATP levels, and the levels of key metabolites in the NAD^+^ synthesis pathway are also elevated (Pinson et al., 2019). The extant research suggests a robust correlation between the aforementioned pathways and the heat tolerance of yeasts. Li et al. (2021) found that differential metabolites of *Kluyveromyces marxianus* were enriched in purine metabolism and pyrimidine metabolism under high temperature stress. Pan et al. (2019) performed metabolomics analysis of *Saccharomyces cerevisiae* after a high temperature incubation, and found that the differential metabolites were associated with a variety of amino acid metabolism (e.g., arginine), energy metabolism, and purine and pyrimidine metabolism.

In order to analyse and explore the effect of oxidative phosphorylation and energy metabolism on the heat tolerance of the strain, a correlation analysis was performed between transcriptomics and metabolomics. The results demonstrated that a variety of metabolites (NAD^+^ and ADP) linked to ATP production and metabolism in the metabolomics exhibited a positive correlation with oxidative phosphorylation DEGs in the transcriptomics. Overall, oxidative phosphorylation provides the cell with a substantial amount of ATP, while NAD^+^ and ADP are closely associated with oxidative phosphorylation and energy metabolism. Consequently, this suggests that the strain can withstand high temperature stress by up regulated energy metabolism (especially oxidative phosphorylation) and increasing ATP production.

## 5 Conclusion

In this study, we investigated a heat tolerant *Pichia kudriavzevii* strain isolated from strong flavor *daqu* of *baijiu*. The strain demonstrated considerable heat tolerance at temperatures up to 50°C, accompanied by significant morphological and structural alterations. To elucidate the molecular mechanisms underlying the heat resistance of this strain, transcriptomics and metabolomics analyses were employed. The results indicated that the strain primarily up-regulated energy metabolism, particularly oxidative phosphorylation, in order to cope with high temperature stress. Furthermore, metabolomics data demonstrated elevated levels of NAD^+^ and ADP, which are closely associated with energy metabolism. The correlation analysis between transcriptomics and metabolomics confirmed a strong positive relationship between these metabolites and differentially expressed genes (DEGs) involved in oxidative phosphorylation. This study provides a molecular basis for understanding the heat resistance mechanism in *baijiu* yeast and offers insights for optimizing production and processing control.

## Data Availability

The raw sequence data presented in this paper have been deposited in the Genome Sequence Archive in the National Genomics Data Center, China National Center for Bio information/Beijing Institute of Genomics, Chinese Academy of Sciences, under accession number CRA022684 and are publicly accessible at https://bigd.big.ac.cn/gsa (accessed on 1 February 2025).
