# Supplementary material for: Combined transcriptomics and metabolomics analyses reveal the molecular mechanism of heat tolerance in Pichia kudriavzevii
Source: Front Microbiol. 2025 Apr 9;16:1572004. doi: 10.3389/fmicb.2025.1572004 (PMC12014439; doi:10.3389/fmicb.2025.1572004)
Supplement: Supplementary file 1 [file Table_1.docx]

Supplementary Material

Table S1 Significantly up-regulated terms in GO enrichment analysis (HT vs. C)

| **Category** | **GO ID** | **Description** | ***p*-adj** |
| --- | --- | --- | --- |
| BP | GO:0006754 | ATP biosynthetic process | 6.88E-03 |
| BP | GO:0009142 | nucleoside triphosphate biosynthetic process | 6.88E-03 |
| BP | GO:0009145 | purine nucleoside triphosphate biosynthetic process | 6.88E-03 |
| BP | GO:0009201 | ribonucleoside triphosphate biosynthetic process | 6.88E-03 |
| BP | GO:0009206 | purine ribonucleoside triphosphate biosynthetic process | 6.88E-03 |
| BP | GO:0009144 | purine nucleoside triphosphate metabolic process | 8.62E-03 |
| BP | GO:0009199 | ribonucleoside triphosphate metabolic process | 8.62E-03 |
| BP | GO:0009205 | purine ribonucleoside triphosphate metabolic process | 8.62E-03 |
| BP | GO:0046034 | ATP metabolic process | 8.62E-03 |
| BP | GO:0009141 | nucleoside triphosphate metabolic process | 1.00E-02 |
| BP | GO:0009124 | nucleoside monophosphate biosynthetic process | 1.00E-02 |
| BP | GO:0009127 | purine nucleoside monophosphate biosynthetic process | 1.00E-02 |
| BP | GO:0009156 | ribonucleoside monophosphate biosynthetic process | 1.00E-02 |
| BP | GO:0009168 | purine ribonucleoside monophosphate biosynthetic process | 1.00E-02 |
| BP | GO:0019693 | ribose phosphate metabolic process | 1.19E-02 |
| BP | GO:0009123 | nucleoside monophosphate metabolic process | 1.23E-02 |
| BP | GO:0009126 | purine nucleoside monophosphate metabolic process | 1.23E-02 |
| BP | GO:0009161 | ribonucleoside monophosphate metabolic process | 1.23E-02 |
| BP | GO:0009167 | purine ribonucleoside monophosphate metabolic process | 1.23E-02 |
| BP | GO:0009150 | purine ribonucleotide metabolic process | 1.54E-02 |
| BP | GO:0009259 | ribonucleotide metabolic process | 1.54E-02 |
| BP | GO:0009117 | nucleotide metabolic process | 1.71E-02 |
| BP | GO:0009152 | purine ribonucleotide biosynthetic process | 1.71E-02 |
| BP | GO:0009260 | ribonucleotide biosynthetic process | 1.71E-02 |
| BP | GO:0046390 | ribose phosphate biosynthetic process | 1.71E-02 |
| BP | GO:0017144 | drug metabolic process | 1.96E-02 |
| BP | GO:0006753 | nucleoside phosphate metabolic process | 2.01E-02 |
| BP | GO:0006163 | purine nucleotide metabolic process | 2.55E-02 |
| BP | GO:0009165 | nucleotide biosynthetic process | 2.71E-02 |
| BP | GO:1901293 | nucleoside phosphate biosynthetic process | 2.71E-02 |
| BP | GO:0072521 | purine-containing compound metabolic process | 3.29E-02 |
| BP | GO:0006164 | purine nucleotide biosynthetic process | 3.32E-02 |
| BP | GO:0055085 | transmembrane transport | 3.57E-02 |
| BP | GO:0072522 | purine-containing compound biosynthetic process | 4.51E-02 |
| BP | GO:1902600 | proton transmembrane transport | 4.51E-02 |
| CC | GO:0005739 | mitochondrion | 3.42E-04 |
| CC | GO:0005740 | mitochondrial envelope | 9.41E-03 |
| CC | GO:0098798 | mitochondrial protein complex | 9.41E-03 |
| CC | GO:0044429 | mitochondrial part | 9.41E-03 |
| CC | GO:0031966 | mitochondrial membrane | 9.41E-03 |
| CC | GO:0005743 | mitochondrial inner membrane | 9.41E-03 |
| CC | GO:0019866 | organelle inner membrane | 9.41E-03 |
| CC | GO:0044455 | mitochondrial membrane part | 9.41E-03 |
| CC | GO:0098800 | inner mitochondrial membrane protein complex | 1.54E-02 |
| CC | GO:0044425 | membrane part | 2.46E-02 |
| CC | GO:0031967 | organelle envelope | 3.85E-02 |
| CC | GO:0031975 | envelope | 3.85E-02 |

Table S2 Differential metabolites associated with heat tolerance (HT vs. C)

| Model | Name | RT [min] | m/z | FC | *p*-value | VIP | Up/Down |
| --- | --- | --- | --- | --- | --- | --- | --- |
| positive | Betaine | 1.381 | 118.08625 | 7.157336133 | 6.17E-13 | 1.104662163 | up |
| positive | Nicotinamide adenine dinucleotide (NAD+) | 1.978 | 664.11567 | 4.289802011 | 7.92E-13 | 1.103357786 | up |
| positive | Adenosine diphosphate (ADP) | 1.978 | 428.03614 | 4.012980592 | 3.47E-10 | 1.101708012 | up |
| positive | Adenosine 3'5'-cyclic monophosphate | 2.765 | 330.05945 | 2.113429293 | 4.90E-07 | 1.089766187 | up |
